# Experience report on surveillance of vancomycin-resistant enterococci

**DOI:** 10.1186/1753-6561-5-S6-P21

**Published:** 2011-06-29

**Authors:** MM Baraldi, CZ Talala, MM Simonetti, CM Santoro

**Affiliations:** 1 Serviço de Controle de Infecção Hospitalar, Hospital Alemão Oswaldo Cruz, São Paulo, Brazil

## Introduction / objectives

Over the years, there has been an increase in infection rates associated with vancomycin-resistant Enterococcus (VRE). Patients with VRE with clinical findings may show signs and symptoms of infection of the urinary tract, bloodstream, wounds, abdominal pain, diarrhea and can progress to septic shock. However, patients may be colonized and have no signs and symptoms, but they represent a strong threat in the spread of bacteria. In the body of the colonized subject, the most important reservoir of VRE is the gastrointestinal tract, especially the colon.

## Methods

This is an experience report retrospective from 2005 to 2010, involving the description of the monitoring of 547 cases of patients who fit the protocols for surveillance of a General Hospital of São Paulo. The 437 cases evaluated by collecting a sample of anal swab for VRE in the research situation Empirical Contact Precautions (at admission) and 110 cases evaluated in Directed Surveillance, including a collection of four samples from each patient, considering the criterion of patient risk.

## Results

In the sample of 437 patients investigated, 2.28% of patients had positive VRE while in the performance of periodic surveillance directed at patients at risk (110 cases), 8.08% showed positive result for the VRE.

## Conclusion

This report demonstrates the prevalence of VRE, considering the microorganisms found in patients who are admitted in Empirical Contact Precautions and the importance of directed surveillance to microorganisms such as VRE, Clostridium difficile and KPC production. Evidence of colonization of patients means it can be empirical precaution and thus provide patient safety by minimizing the risk of nosocomial infection.

## Disclosure of interest

None declared.

